# Point Defects in Two-Dimensional Indium Selenide as Tunable Single-Photon Sources

**DOI:** 10.1021/acs.jpclett.1c02912

**Published:** 2021-11-04

**Authors:** Mattia Salomone, Michele Re Fiorentin, Giancarlo Cicero, Francesca Risplendi

**Affiliations:** † Dipartimento di Scienza Applicata e Tecnologia, Politecnico di Torino, corso Duca degli Abruzzi 24, 10129 Torino, Italy; ‡ Center for Sustainable Future Technologies, Istituto Italiano di Tecnologia, via Livorno 60, 10144 Torino, Italy

## Abstract

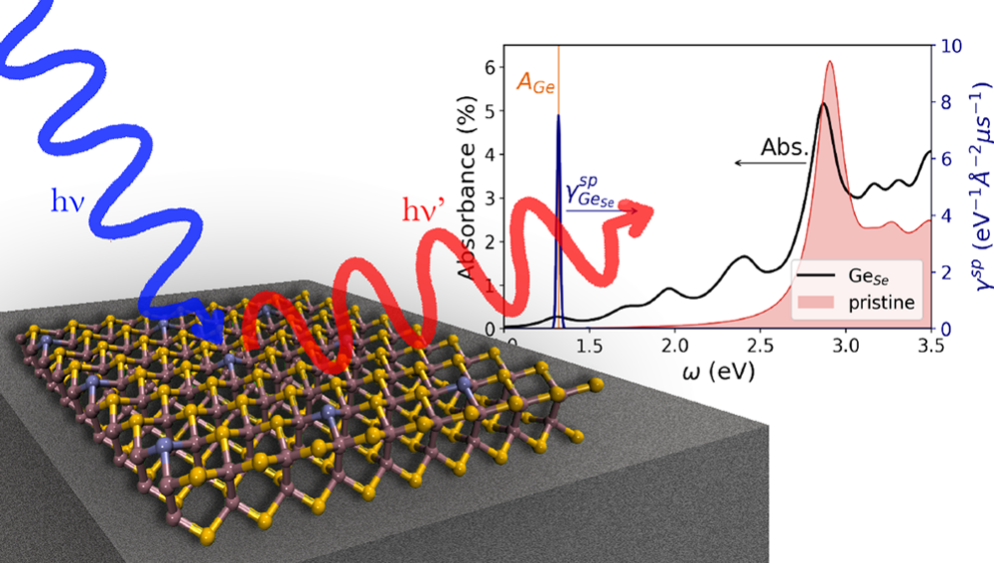

In the past few years remarkable interest has been kindled by the development of nonclassical light sources and, in particular, of single-photon emitters (SPE), which represent fundamental building blocks for optical quantum technology. In this Letter, we analyze the stability and electronic properties of an InSe monolayer with point defects with the aim of demonstrating its applicability as an SPE. The presence of deep defect states within the InSe band gap is verified when considering substitutional defects with atoms belonging to group IV, V, and VI. In particular, the optical properties of Ge as substitution impurity of Se predicted by solving the Bethe–Salpeter equation on top of the GW corrected electronic states show that transitions between the valence band maximum and the defect state are responsible for the absorption and spontaneous emission processes, so that the latter results in a strongly peaked spectrum in the near-infrared. These properties, together with a high localization of the involved electronic states, appear encouraging in the quest for novel SPE materials.

Photonic technologies are considered the most promising future steps for industrial and scientific innovation. With wide-reaching applications, from sensors for biomedical imaging to superfast computers and ultrasecure communication, photonics may provide solutions to many challenges in our daily lives.^1−4^ In particular, on-demand and truly scalable sources of indistinguishable single photons, otherwise called single-photon emitters (SPEs), represent fundamental building blocks for optical quantum technologies.^5−8^ Given the importance of quantum light in both remote quantum computing and quantum communication, the search for reliable and efficient SPEs is a thriving open field. In fact, existing lasers are inherently unsuitable to generate single photons, and new compact and more reliable single-photon sources that could be easily integrated into quantum optics applications are needed.^8^

Promising structures behaving as SPEs consist of atom-like systems that intrinsically emit one photon at a time. These have been obtained by generating isolated color centers in three-dimensional and two-dimensional semiconductor materials such as diamond,^9−12^ and hexagonal boron nitride h-BN,^11−13^ as well as in one-dimensional materials, such as carbon nanotubes^14^ and InP nanowires,^15^ and zero-dimensional materials, such as GaAs and InGaAs quantum dots.^16,17^ To achieve efficient single-photon emission, it is mandatory to avoid electron/hole recombination, which ultimately implies that the electron energy levels of the atom-like structure, for example arising from substitutional defects, must be deep in the host matrix band gap. In this respect, materials with wide energy gaps are the most suitable candidates to be used as matrixes for single-photon emitters. In addition, sharp optical transitions and narrow emission peaks require that the impurity orbitals do not hybridize strongly with the host material^18^ orbitals; that is, the impurity states must be flat without showing dispersion in reciprocal space.

Transition metal dichalcogenides (TMDs) have shown unprecedented features which make them optimal materials for single-photon sources.^3^ TMDs are characterized by a layered structure held together by weak van der Waals forces. These can be reduced to a single layer by simple exfoliation or other means.^19−21^ The 2D systems obtained in this way have the advantage of exhibiting strong light–matter interaction and large exciton binding energy. Optical confinement and emission can be further tailored either by stacking more than a single layer or by applying external electrostatic^22^ or strain^3,23,24,^ fields. Among the others, single-photon emitters have been obtained with WSe_2_^25,26^ and WS_2_,^27^ and narrow emission lines of localized centers have been found in MoSe_2_^28^ too. Recently, single-photon emission was discovered in other metal chalcogenides, such as GaSe. In this compound, local deformations of the crystal trap excitons^7^ are responsible for SP emission.^3,24,29^ These discoveries have stimulated the search for better single-photon sources in other two-dimensional metal chalcogenides.

In this Letter, we propose the use of InSe as SPE and show how to tailor its optical emission by introducing substitutional defects in its monolayer structure. The focus on this material has been stimulated by experimental evidence that as-grown InSe presents selenium vacancies at its surface and that these defects can be passivated^30^ by introducing different types of heteroatoms (Se substitutional defects, X_Se_). Further, several studies^3,31^ have evidenced that 2D InSe, whose structure and band diagram are reported in Figure 1, is characterized by a reduced valley hybridization and a relatively large band gap. These features are expected to give rise to little dispersion of impurity states and consequently to originate single-atom-like behavior of substitutional defects. As such, the InSe monolayer presents some important prerequisites required to effectively function as an SPE host matrix. The strong light–matter interaction reported^32^ for InSe may further boost optical SPE efficiency. In this study we consider the passivation of selenium vacancies with atomic species of the IV, V, and VI group of the periodic table (namely Ge, As, P, N, S, and O) and calculate the optoelectronic properties of the resulting structures by means of *ab initio* simulations. Our results highlight that some of the proposed structures show sharp optical transitions involving flat atom-like impurity states, which could be stimulated on demand, for example by coupling the two-level system to a resonant cavity.^33−36^ Further, it is shown that the emitted frequency can be tuned by changing the depth and localization of the defect level by changing the atomic species used to saturate the vacancy sites.

**Figure 1:**
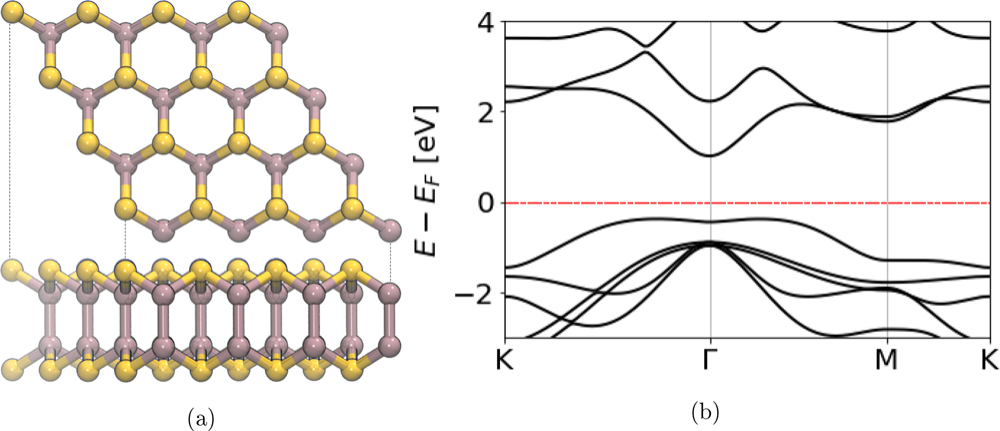
Left panel: top and side views of an InSe monolayer. Se atoms are represented in yellow, while In atoms are in gray. Right panel: InSe band structure calculated at the DFT-PBE level.

Calculations are based on spin-polarized DFT as implemented in the Quantum Espresso package.^37,38^ We employ ultrasoft pseudopotentials^39^ to describe the electron–ion interaction and the gradient-corrected Perdew–Burke–Ernzerhof (PBE) functional^40^ to describe the exchange–correlation effects. Computational details can be found in the Supporting Information. To overcome the limitations of DFT in the estimation of the electronic band gap and optical properties, we apply many-body perturbation theory (MBPT) to systems that appear most promising after the DFT screening. The band structure is corrected within the G_0_W_0_ approximation, and the optical absorption is studied by solving the Bethe–Salpeter equation (BSE).^41^ All post-DFT calculations are performed by means of the YAMBO code.^42^ Details on the MBPT convergence procedure and parameters are reported in the Supporting Information.

We first compare the impurity defect formation energies with the vacancy formation energy, *E*_Form_, computed in the low-concentration limit as defined in the Supporting Information. All X_Se_ species considered in this study, except for germanium and nitrogen, yield *E*_Form_ values smaller than the vacancy formation energy along the whole range of variability of the In and Se chemical potentials (*cf.* the Supporting Information). The *E*_Form_ of Ge_Se_ and N_Se_ defects are comparable to the cost of generating a vacancy in the lattice. Because vacancies are widely observed in experimental samples,^43−45^ the obtained *E*_Form_ values point at technologically achievable substitutions. As a general trend we observe that *E*_Form_ decreases along the columns of the periodic table (from the second to the third row) and decreases along the rows (from right to left): impurities with dimensions closer to Se are more favorable (see Figure S1 of the Supporting Informations). Interestingly, O and S substitutions show negative formation energies confirming the experimentally observed tendency of InSe to spontaneously incorporate oxygen when exposed to air.^30,43,44^

The modifications of the electronic properties of InSe induced by vacancies and substitutional impurities are analyzed in terms of band structure, density of states (DOS), projected density of states (pDOS), and spatial distribution of the charge densities associated with defect states. In addition, we report the positions of defect levels (*E*^d^) referred to the Fermi level.

InSe containing selenium vacancies presents fully occupied defect states (*i.e.*, donor states) in the energy gap, as also shown in the band diagram reported in Figure 2a (left panel). These one-electron states have predominantly P_Se_ character with some hybridization with In p orbitals, while the lower conduction band has mainly S_In_ and P_In_ character. Considering the total DOS and the projections on Se and In species, also reported in Figure 2a, it is worth noting that both Se and In atoms contribute to the defect states. Moreover, Figure 2a (right panel) shows that the charge density associated with these states is spatially localized around the Se vacancy and equally distributed around surrounding atoms (both Se and In). In this structure, the defect state coincides with the highest occupied state, and it is localized below the Fermi energy. The corresponding lowest electronic transition has defect level-conduction band minimum (D-CBm) character, and it occurs at approximately 1 eV.

**Figure 2:**
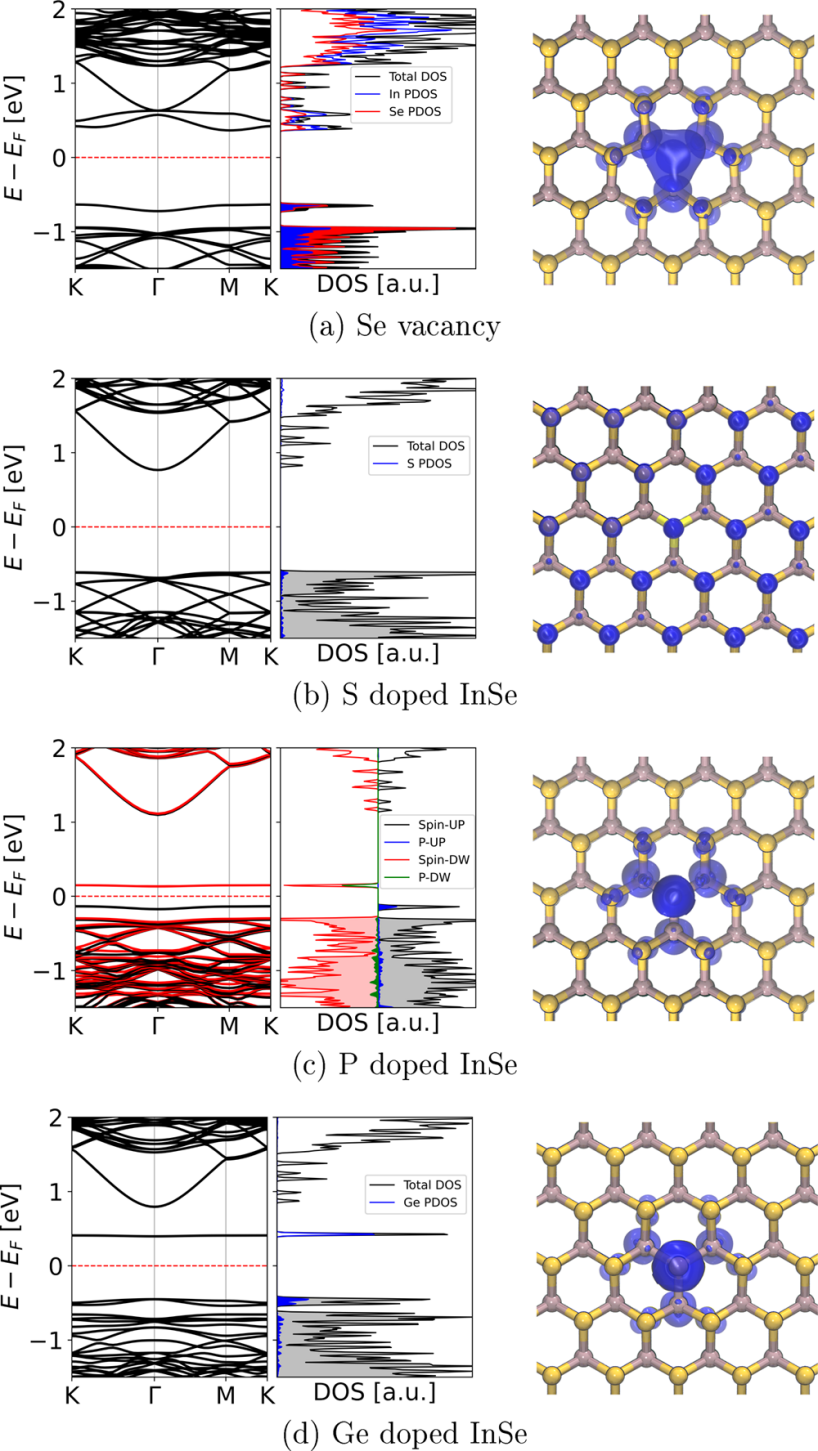
Band structures, density of states, and defect level spatial localization of InSe monolayer computed in 4 × 4 supercells with different lattice modifications: In panel (a) it is possible to see a purely donor defect state. In panel (d) the defect level is completely empty (acceptor state), whereas in panel (c) the defect level is split because of spin polarization. In panel (b), instead, the defect level is degenerate with the VB.

When Se vacancies are saturated with group VI species (O and S), vacancy defect states are pushed down in energy and become degenerate with states of InSe valence band (VB). This is an indication that the bonds between these atoms and the nearby In atoms are somewhat similar to that of In–Se bonds, thus resulting in a complete saturation of the dangling bonds around the vacancy site. Indeed, in this case the highest occupied state is delocalized on the whole 2D layer and does not show localization typical of impurity states. This is clearly visible in the first panel of Figure 2b. Further, this finding shows that even when present as residual impurity after InSe growth or when used to saturate possible residual vacancy sites, group VI species do not have detrimental effects on InSe optical properties because they do not originate trap states in the gap but rather eliminate them.

Figure 2c shows the DOS/pDOS, the band diagram (left panel), and the electron density of the highest occupied state (right panel) when phosphorus substitutes selenium atoms. Other group V species yield similar results in terms of electronic structure (see the values reported in Table 1). In particular, P originates defect states in the InSe band gap characterized by different spin. The unoccupied state results at ∼1.3 eV below the bottom of the conduction band, whereas the occupied state is ∼0.4 eV above the valence band. Two low-energy transitions can be defined in this case, one corresponding to D-CBm and the other one related to VBM-D, which are characterized by *E*_Trans_ = 1.29 and 0.45 eV, respectively.

**Table 1:** DFT Results on Vacancy and Substitutional Defects in Monolayer InSe^a^

defect type	*E*^d^ (eV)	transition type	*E*_Trans_ (eV)
Se vac.	–0.64	D-CBm	1.00
Ge_Se_	0.42	VBM-D	0.86
N_Se_	–0.21/0.23	VBM-D/D-CBm	0.50/1.29
P_Se_	–0.14/0.15	VBM-D/D-CBm	0.45/1.29
As_Se_	–0.11/0.13	VBM-D/D-CBm	0.40/1.27
O_Se_		VBM-CBm	1.31
S_Se_		VBM-CBm	1.45

a *E*^d^ reports the position of the defect state with respect to the Fermi energy. In the case of oxygen and sulphur, which generate defects hybridized with valence band states, *E*^d^ has not been calculated. For group IV atoms, we report the two values given by the splitting of the defect states due to spin-polarization. The third column specifies the lowest-energy electronic transitions: VBM-D (D-CBm) marks a transition from the valence band maximum to the defect level (from the defect level to the conduction band minimum). *E*_Trans_ represents the corresponding transition energies, computed at the DFT level.

Results for Ge impurity substituting Se are shown in Figure 2d, as representative of the group IV species. The DOS presents a narrow peak within the InSe band gap as a result of an almost perfectly flat defect level. At variance with the cases of Se vacancy and group V impurities, Ge defect states fall closer to the CB and well above the Fermi level, thus behaving as acceptor states (*E*^d^ = 0.42 eV). The lower electronic energy transition connects VBM-D states and occurs at *E*_Trans_ ≈ 0.86 eV.

Notably, when group IV species substitute Se atoms in InSe monolayer, the contribution of the X_Se_ atom to the DOS of InSe top VB (tVB) and CB is almost negligible (see left panel of Figure 2c), while, in the case of group V subsitutions, the tVB is significantly affected by the presence of the defect (left panel of Figure 2d). In fact, for Ge_Se_ the highest occupied band shows such a large contribution from Ge orbitals and low dispersion in *k*-space that may be as well considered a Ge-induced defect state. Both for group IV and group V substitutions, the electronic states observed within the band gap show dominant pDOS contributions from the heteroatoms. Moreover, the electron density pertaining to these defects is strongly localized around the impurity site (see right panels of Figure 2c,d). This finding confirms that these impurities in InSe monolayer represent promising structures for SPEs.

Considering the extremely small dispersion in energy of the resulting level, its depth within the band gap, and clear acceptor character of Ge modified InSe monolayer, we make an in-depth analysis of the optical features of this system. The study of the stability of Ge_Se_ in different charge states^46,47^ points out that the most representative germanium substitution in intrinsic InSe is charge neutral (*cf*. the Supporting Information). For this reason, our following investigations focus on neutral Ge_Se_ defects. We first obtain its quasi-particle (QP) band structure within the G_0_W_0_ approximation and then predict its absorption and emission spectra. In Figure 3a we report the QP bands of the Ge_Se_-defected monolayer, aligned to the VBM and CBm of the pristine InSe monolayer (red-shaded regions), for comparison. It is possible to notice that VBM-D is widened to 2.11 eV and that the tVB of Ge_Se_ is found at higher energies than the pristine VBM. This result supports the previous pDOS analysis, Figure 2d, suggesting that this low-dispersion top valence band is not bulk-like, but rather another Ge-induced defect level. As a consequence of the upshift in energy of tVB in Ge_Se_, the gap between the VBM and the conduction band above the defect level is reduced to 2.67 eV, while in pristine InSe the band gap is computed to be 2.92 eV, in very good agreement with the literature.^48,49^ The QP energies obtained within the G_0_W_0_ approximation are used to build and solve the BSE and obtain the optical properties of the Ge_Se_-defected monolayer. In Figure 3b we report the absorbance of the defected monolayer (thick black line) and of the pristine InSe monolayer (red-shaded region), as defined in the Supporting Information. The highest absorbance peak of the defected monolayer falls in the close proximity of the absorption peak of pristine InSe (∼2.88 eV), but several new peaks appear at lower energies. Clearly, the intensities of these additional peaks depend on the simulated defect concentration, *i.e.*, one substitutional defect per 4 × 4 supercell, corresponding to a defect surface density of ∼0.45 nm^–2^. However, we have checked that the chosen supercell ensures the suppression of long-range interactions between periodic replicas of the defect, so that the positions of the additional peaks are not affected by finite-size effects. The first absorption peak is due to a bright exciton, *A*_Ge_, at  eV, whose electronic transitions involve the tVB and the defect state *D*, marked in orange in Figure 3a. *A*_Ge_ binding energy,  eV, is particularly high, as is common in low-dimensional materials.^50−52^

**Figure 3:**
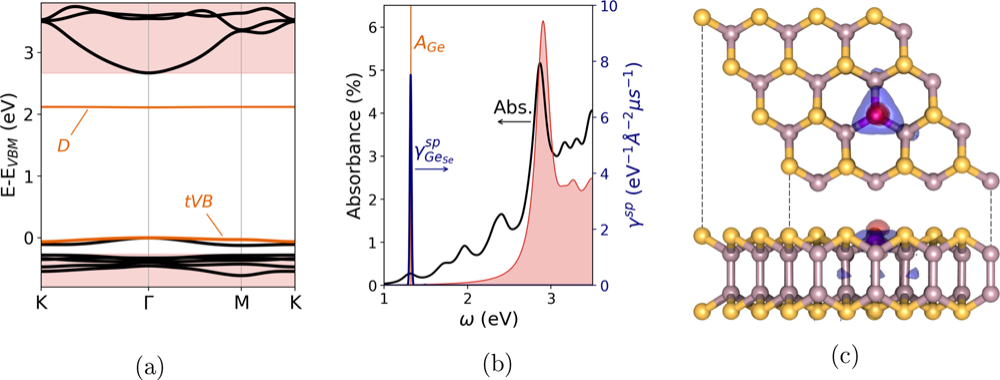
(a) G_0_W_0_ quasi-particle band structure of the Ge_Se_ defected InSe monolayer in the 4 × 4 supercell, thick black lines. The shaded red regions mark the valence and conduction bands of pristine InSe monolayer, aligned to the Ge_Se_ VBM. (b) Absorbance of pristine InSe monolayer (shaded region) and Ge_Se_-defected (thick black line). The vertical line marks the *A*_Ge_ exciton state. The blue peak represents the spontaneous emission rates . Absorbance and emission values for Ge_Se_ InSe refer to the simulated 4 × 4 supercell. (c) Average electron (red) and hole (blue) densities for exciton *A*_Ge_ in Ge_Se_ InSe.

Following the excitation, we expect the defected system to relax to the lowest-energy exciton state, so that the zero-phonon line (ZPL)^53^ of the emission spectrum of Ge_Se_ will originate from the radiative recombination of *A*_Ge_. We can gain some insight into this process by computing the spontaneous emission rate γ^sp^ (per unit energy, per unit surface) exploiting the Roosbroeck–Shockley relations,^54^ neglecting exciton–phonon coupling. In Figure 3b, with the blue line, we report the spontaneous emission spectrum of the 4 × 4 Ge_Se_ supercell, . Because of the gap in energy between higher-energy exciton states and the lowest-energy one, only exciton *A*_Ge_ contributes to the ZPL. As in the case of the absorption spectrum, the emission peak intensity depends on the chosen defect concentration, while its position is not affected by the finite-size effect. Independently of the defect density, these results point out that Ge_Se_ monolayers will present an emission of photons with energy  eV (neglecting exciton–phonon coupling), originating from spatially isolated sources. Indeed, in Figure 3c the red and blue isosurfaces report the average densities of the electron and hole, respectively, bound in *A*_Ge_, *cf*. the Supporting Information. It is possible to notice that exciton *A*_Ge_ is spatially localized around the defect, ensuring that the studied Ge substitutions are promising candidates of isolated single-photon emitters.

In conclusion, in this Letter, we studied the properties of point defects in InSe obtained through the substitution of selenium atoms with species belonging to groups IV (Ge), V (N, P, and As), or VI (O and S) of the periodic table. The computed formation energies and the analysis of the corresponding band structures show that S and O substitutions can be exploited to saturate dangling bonds (Se vacancy defects) spontaneously present in the experimental sample and cure the 2D layer in a way that does not modify the optical emission or absorption of the pristine monolayer. N, P, As, and Ge substitutions of Se generate defect levels that are well distinct both from the VB and the CB of InSe and allow for new electronic sharp transitions. We have applied MBPT to Ge_Se_ defected monolayer and obtained its absorbance and spontaneous emission spectrum by solving the BSE. We have shown that the defect states in this material realize a single-atom-like system with a low-energy absorption peak due to electronic transitions between the tVB and the defect state. Our results highlight that the proposed Se substitutions, in particular Ge_Se_, can be attractive for SPE because they possess strong spatial localization; their formation energies are not prohibitive for their employment; and, in particular, the depth of the obtained defect levels guarantees their thermal stabilities and enables electronic transitions which result in emission spectra, which may be stimulated on demand.

## Supporting Information Available

Details on the DFT, G_0_W_0_, and BSE calculations; absorbance and emission rate definitions (PDF)
