# Supplementary material for: Point Defects in Two-Dimensional Indium Selenide as Tunable Single-Photon Sources
Source: J Phys Chem Lett. 2021 Nov 4;12(45):10947–52. doi: 10.1021/acs.jpclett.1c02912 (PMC8607502; doi:10.1021/acs.jpclett.1c02912)
Supplement: Supplementary file 1 — jz1c02912_si_001.pdf [file jz1c02912_si_001.pdf]

Supporting Information for

Point defects in two-dimensional Indium Selenide as Tunable Single Photon Sources

Mattia Salomone[1], Michele Re Fiorentin[2], Giancarlo Cicero[1] and Francesca Risplendi[1]
[1] *Dipartimento di Scienza Applicata e Tecnologia, Politecnico di Torino, corso Duca degli Abruzzi 24, 10129 Torino, Italy*
[2] *Center for Sustainable Future Technologies, Istituto Italiano di Tecnologia, via Livorno 60, 10144 Torino, Italy*

1 DFT computational approach

All simulations are based on the density functional theory (DFT) as implemented in the Quantum Espresso package [1, 2]. The Kohn-Sham equations are solved using ultrasoft pseudopotentials to describe electron-ion interactions, employing the gradient corrected Pendew, Burke and Ernzerhof (PBE) functional [3] to represent the exchange correlation effects, and expanding the electronic wavefunctions in plane waves (PWs). For all calculations we adopted a PW cutoff energy of 45 Ry for the wavefunctions and of 450 Ry for the charge density and potentials. The Brillouin Zone (BZ) is sampled employing a (6 6 1) Monkhorst-Pack (MP) grid [4] in the case of the 2D InSe unit cell, and a reduced MP grid in the case of the supercells. All structures were relaxed by minimizing the atomic forces; convergence was assumed when the maximum component of the residual forces on the ions was smaller than 10^{-4} Ry/bohr. Under these computational conditions we found a value for the equilibrium lattice parameter **a** of 4.09 Å in agreement with the experimental value [5].

2 Spin-Orbit coupling

We evaluated the impact of including Spin Orbit Coupling (SOC) in the calculation of InSe properties and we report InSe monolayer band structure considering SOC and compare to the one obtained without including it (figures S1a and S1b). Band diagrams were calculated under convergence conditions: cutoff energy of 45 Ry, grid k-points (6 6 1) and a=4.09 Å. It can be noticed, in figures S1a and S1b, the so called sombrero-shaped dispersion around the point Γ, which is given by the presence of two maxima around that k-value and an almost flat VB between them. This feature leads to an enhancement of the probability of electronic transitions between Valence Band (VB) and Conduction Band (CB). An analysis of the band dispersion in figure S1b (where SOC is considered) highlights that the two band structures are very similar, particularly around the top of the valence band and the bottom of the conduction band, which is the most relevant part when interested in photons emission. Bandgaps are also very similar, in agreement with [6]. In conclusion, in view of the negligible differences observed in the predicted properties of InSe monolayer, all results in this article have been obtained without including spin-orbit coupling.

3 Defect formation energy and supercell test

In order to ensure that the results of the calculations accurately represent an isolated defect, the supercell must be large enough so that impurities of adjacent cells (replicas) do not interact with each other. To find the optimal supercell size, we analyzed the variation of the formation energy of the defects as a function of the dimensions of the system, together with the variation of the band structure.

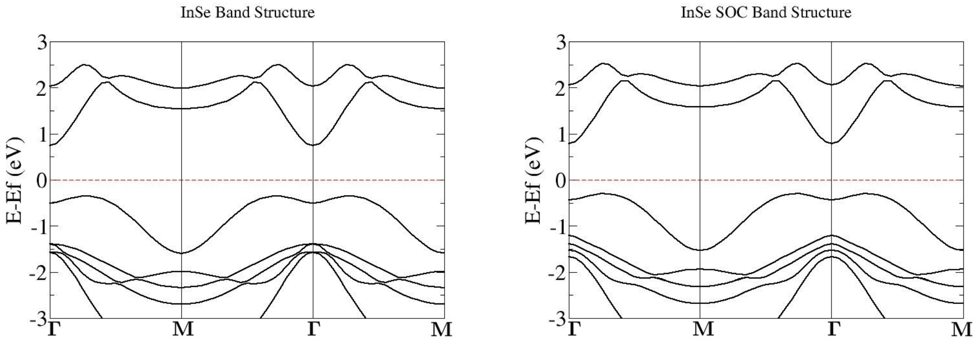

(a) (b)

Figure S1: InSe DFT-PBE band structure considering the Spin-Orbit Coupling (a) and using scalar relativistic pseudopotentials (b).

Within the Density Functional Theory calculations, defect formation energies E_{FORM}, can be obtained via total energy calculations of supercells containing the defect. In particular, for InSe monolayer containing defect X, E_{FORM} has been calculated as follows [7–9]

$$E_{FORM}\left[X\right] = E_{\mathrm{TOT}}\left[X\right] - E_{\mathrm{InSe}}^{\mathrm{TOT}} + n_{\mathrm{In}}\mu_{\mathrm{In}} + n_{\mathrm{Se}}\mu_{\mathrm{Se}} - \sum_{X} n_{\mathrm{X}}\mu_{\mathrm{X}}, \tag{S1}$$

where $E_{\mathrm{InSe}}^{\mathrm{TOT}}$ is the total energy of the pristine supercell and $E_{\mathrm{TOT}}\left[X\right]$ is the energy of the defect containing supercell. In the case of substitutional impurities, X indicates the atomic species introduced in the InSe layer, while n_{X} and μ_{X} are the number of X atoms added to the system and their chemical potential in the reference bulk phases, respectively. In Table S1 we report the phases considered for each atom X. n_{Se} and n_{In} are the number of Se and In atoms removed from the layer and substituted by X species. μ_{Se} and μ_{In} are the chemical potentials of selenium and indium, respectively, and they can be expressed as $\mu_{\mathrm{Se(In)}} = \mu^{0}_{\mathrm{Se(In)}} + \Delta\mu_{\mathrm{Se(In)}}$, where the reference potential $\mu^{0}_{\mathrm{Se(In)}}$ is taken from the stable bulk phase (trigonal selenium, space group $P3_121$ and I4/mmm tetragonal indium). By definition $\mu_{\mathrm{Se(In)}} \leq \mu^{0}_{\mathrm{Se(In)}}$ while the thermodynamical equilibrium requires $\mu_{\mathrm{InSe}} = \mu_{\mathrm{Se}} + \mu_{\mathrm{In}}$. μ_{InSe} is taken as the energy of the InSe monolayer unit cell. Considering $\Delta\mu = \Delta\mu_{\mathrm{Se}} - \Delta\mu_{\mathrm{In}}$ [7, 10] and taking into account the equilibrium relation between chemical potentials, it is possible to rewrite the equation (S1) as

**Defect atom**	**Lat. Parameter/ Bond distance (Å)**	**Reference phase**
Ge	5.76	α-germanium, Fd$\overline{3}$m
P	5.87	Black phosphorus, Cmce
As	4.21	Gray arsenic, R$\overline{3}$m
S	13.96	Octasulfur, P2/c
N	1.11	N_2 molecule
O	1.23	O_2 molecule

Table S1: Phases considered to calculate the chemical potential μ_{X} of each impurity along with their equilibrium lattice parameters (for oxygen and nitrogen we reported the bond distance). For solid reference phases the crystal space group is also reported.

$$E_{FORM}\left[X\right] = E'_{\mathrm{TOT}}\left[X\right] - \frac{1}{2}\left(n_{\mathrm{Se}} - n_{\mathrm{In}}\right)\Delta\mu - \sum_{X} n_{\mathrm{X}}\mu_{\mathrm{X}} \tag{S2}$$

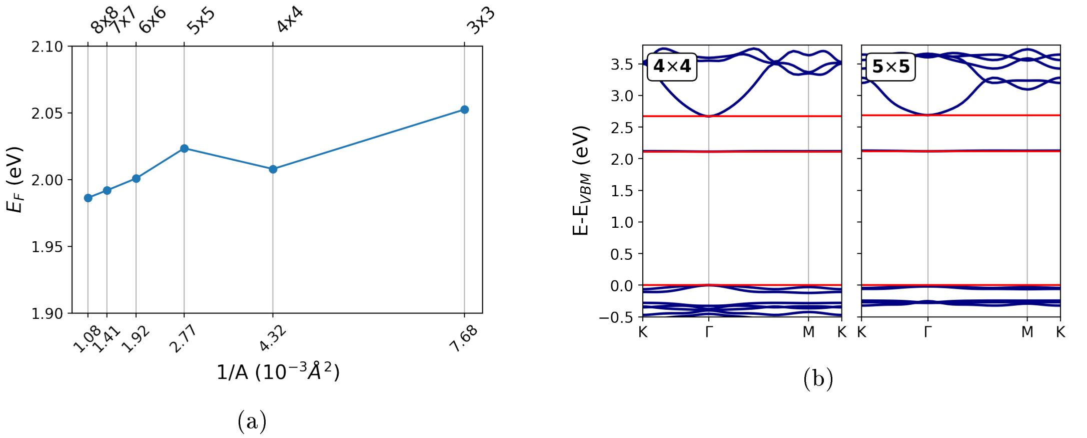

(a) (b)

Figure S2: (a) Formation energy of Ge_{Se} defect computed in supercells with different sizes. (b) Quasiparticle bandstructure of Ge_{Se} computed in the 4×4 (left panel) and 5×5 (right panel) supercells.

where $E'_{\mathrm{TOT}} = E_{\mathrm{TOT}}[X] - E^{\mathrm{TOT}}_{\mathrm{InSe}} + 1/2(n_{\mathrm{Se}} - n_{\mathrm{In}})(\mu^0_{\mathrm{In}} - \mu^0_{\mathrm{Se}}) - 1/2(n_{\mathrm{Se}} + n_{\mathrm{In}})\mu_{\mathrm{InSe}}$. Introducing the enthalpy of formation of an InSe layer $\Delta H_{\mathrm{InSe}} = \mu_{\mathrm{InSe}} - \mu^0_{\mathrm{Se}} - \mu^0_{\mathrm{In}}$ it is possible to introduce meaningful constrains on the $\Delta\mu$ parameter, so that

$$\Delta H_{\mathrm{InSe}} \leq \Delta\mu \leq -\Delta H_{\mathrm{InSe}}. \tag{S3}$$

In Figure S2a we report the formation energy of the neutral Ge_{Se} obtained in various supercells with respect to the inverse of the supercell area. It is possible to notice that the value of E_{FORM} varies by less than 50meV in passing from a 4×4 to an 8×8 supercell. Moreover, by analyzing the bandstructure and the dispersion of the defect states in larger supercells, we confirmed that the results obtained in the 4×4 supercell are not affected by finite-size effects at the many-body level. In Fig. S2b we report the quasiparticle bandstructures computed within the G_0W_0 approximation (see following section) in the 4×4 (left panel) and 5×5 (right panel) supercells. Apart from band folding, the quasiparticle bandstructure undergoes negligible changes in passing from 4×4 to 5×5 supercells. In particular, the defect state correctly shows very little dispersion already in the 4×4 one, which then confirms suitable for the description of an isolated defect.

Figure S3a shows the obtained formation energies for the different substitutional impurities and for Se vacancy.

The substitutional Ge defect Ge_{Se} has been studied in the neutral, +1, +2 and -1 charge states. The energy of formation of the defect with charge q has been computed as [11]

$$E_{FORM}[X^q] = E'_{\mathrm{TOT}}[X^q] - \frac{1}{2}(n_{\mathrm{Se}} - n_{\mathrm{In}})\Delta\mu - \sum_X n_{\mathrm{X}}\mu_{\mathrm{X}} + q(\epsilon_F + E_{\mathrm{VBM}}), \tag{S4}$$

where E_{VBM} is the valence band maximum (VBM) of pristine InSe with respect to the vacuum level and ϵ_F is the Fermi level with respect to VBM. $E'_{\mathrm{TOT}}[X^q]$ is obtained by computing $E_{\mathrm{TOT}}[X^q] - E^{\mathrm{TOT}}_{\mathrm{InSe}}$ on supercells whose lattice parameters a and c are uniformly scaled, and extrapolating to the inifinite-volume limit [12]. Thanks to the uniform scaling of all three directions, a converged result is recovered without correction terms in Eq. S4.

In Figure S3b we report the stability diagram of Ge_{Se} charged defects as a function of the Fermi level and $\Delta\mu$. When considering intrinsic InSe ($\epsilon_F = E^{\mathrm{gap}}/2$, dashed horizontal line), the defect that is stable in the largest variability range of the In and Se chemical potentials ($\Delta\mu$) is the neutral $[Ge_{Se}]^0$, while the positively charged $[Ge_{Se}]^{+1}$ is stable for $\epsilon_F = E^{\mathrm{gap}}/2$ only in a minor region around ΔH_{InSe}. Negatively charged and doubly positively charged Ge are never stable for $\epsilon_F = E^{\mathrm{gap}}/2$. For these reasons we have focused our study on neutral germanium substitution which is the most representative defect in intrinsic InSe.

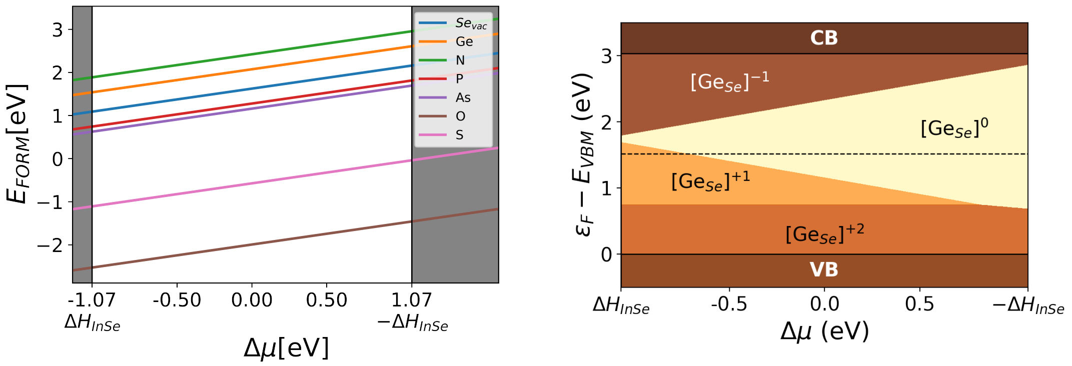

(a) (b)

Figure S3: (a) Formation energies of substitutional and vacancy defects in InSe as a function of $\Delta\mu$ (equation S2). Shaded areas mark the excluded regions according to equation S3. (b) Stability regions of Ge_{Se} defects with charge -1 $[Ge_{Se}]^{-1}$, 0 $[Ge_{Se}]^{0}$, +1 $[Ge_{Se}]^{+1}$ and +2 $[Ge_{Se}]^{+2}$ as a function of $\Delta\mu$ and Fermi level. The dashed horizontal line marks the Fermi level of intrinsic InSe.

4 Convergence details of G_0W_0 and BSE calculations

Quasi-particle (QP) bandstructures are obtained within the one-shot G_0W_0 approximation, while optical and excitonic properties are computed by solving the Bethe-Salpeter equation (BSE). To identify the parameters suitable for converged calculations of the defected monolayers in 4×4 supercells, we first determined the converged parameters for the pristine InSe unit cell and then properly rescaled them to the supercell system.

In all many-body calculations, we considered 25 Å of vacuum along the direction (z) perpendicular to the monolayer. The Coulomb potential was truncated along z and integrals over the Brillouin zone were performed according to the random integration method (RIM) [13,14]. Adopting the QP bandgap at the Γ point as convergence parameter and a 50 meV threshold, we first performed a test on the k-point grid, as reported in fig. S4a. Given the chosen threshold, we deemed satisfactorily converged the result obtained with 61 k-points in the irreducible Brillouin zone, provided by the $24 \times 24 \times 1$ mesh. Convergence of the bandgap with respect to the number of empty bands included in the computation of the correlation self energy Σ_c is reported in fig. S4b. The adoption of Bruneval-Gonze terminators [15] results in a very fast convergence of the bandgap with the number of bands, so that, within our adopted threshold, we chose 400 bands. In fig. S4c we report the results obtained by varying both the number of bands N_b included in the calculation of the dielectric function $\varepsilon_{\mathbf{GG'}}$ and the energy cutoff $E_{\mathbf{G}}$ on its matrix size. The asymptotic value of the bandgap, obtained for $E_{\mathbf{G}} \gtrsim 20$ Ry and $N_b \gtrsim 3000$, and the 50 meV threshold (marked by the shaded region) allow us to choose the lower values $N_b = 100$ and $E_{\mathbf{G}} = 5$ Ry, in view of the more demanding calculations in the 4×4 supercell. In fig. S4d we report the final G_0W_0 QP bandstructure of pristine InSe. In particular, we mark the direct bandgap at Γ, $E^{(d)}_{\text{gap}} = 2.99$ eV, and the indirect bandgap, $E^{(i)}_{\text{gap}} = 2.92$ eV, between the conduction band minimum at Γ and the valence band maximum at a k-point intermediate between Γ and M. The value of $E^{(i)}_{\text{gap}}$ is in perfect agreement with previous literature results on the bandgap of InSe when spin-orbit coupling is neglected [16,17].

The optical properties reported in this work assume an in-plane polarization of the electric field at 45° with respect to the x axis (horizontal in fig. 1(a) in the manuscript). In fig. S5a we report the imaginary part of the macroscopic dielectric function computed on different grids. The exchange-correlation contribution to the BSE kernel was built within the static G_0W_0 approximation, by computing the static screened Coulomb potential. It is possible to notice that a much denser grid, at least $32 \times 32 \times 1$, is necessary to obtain a converged spectrum. We also assessed the convergence of the static screening by carrying out a series of calculations with increasing number of bands and cutoff on the coarser $24 \times 24 \times 1$ grid, fig. S5b. The dielectric screening can be computed by using a smaller number of bands, $N_b^{\text{BSE}} = 40$, and a lower cutoff, $E_{\mathbf{G}}^{\text{BSE}} = 1$ Ry.

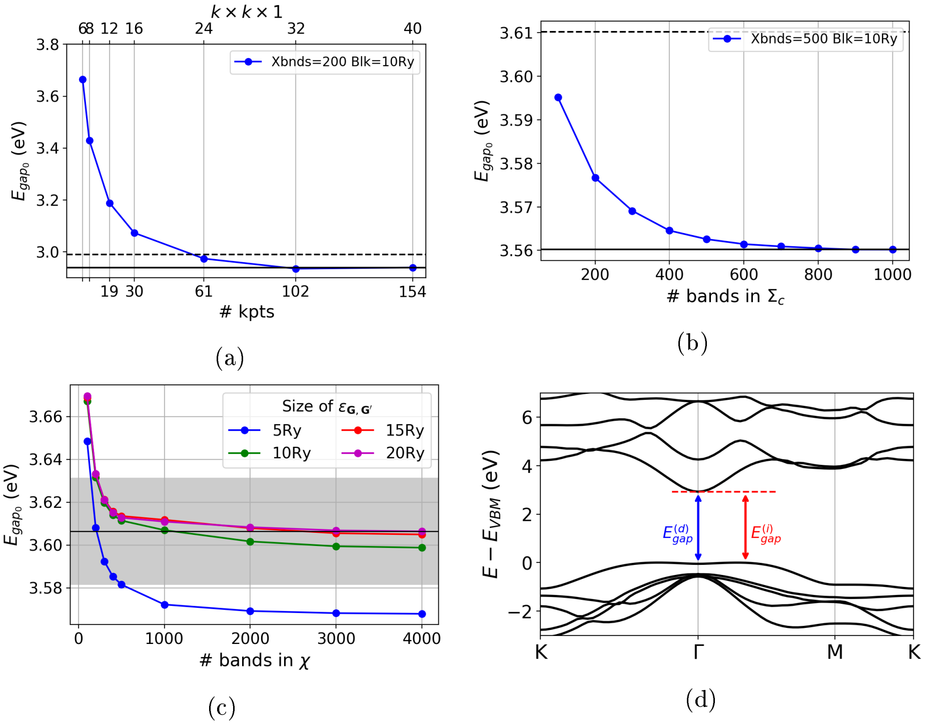

Figure S4: Convergence tests on G_0W_0 calculations. (a) Variation of the QP bandgap at the Γ point with respect to the k-point grid. (b) Variation of the QP bandgap at the Γ point with respect to the number of bands included in the calculation of Σ_c. (c) QP bandgap at the Γ point obtained on the coarse $6 \times 6 \times 1$ grid by varying the number of bands and the energy cutoff of the dielectric screening. (d) QP bandstructure of pristine InSe.

5 Absorbance and spontaneous emission rate

For 2D materials, the macroscopic dielectric function necessarily tends to the value of the surrounding vacuum, thus yielding a vanishingly small imaginary part. For this reason, it is more useful to employ the polarizability α^{2D} defined as

$$\alpha^{2D} = \chi L = \frac{\varepsilon - 1}{4\pi} L, \tag{S5}$$

where χ is the dielectric susceptibility and L is the dimension of the simulation cell in the non-periodic direction (the out of plane direction in our case). This way, we obtain the imaginary part of the polarizability as

$$\mathrm{Im}(\alpha^{2D}) = \frac{\mathrm{Im}(\varepsilon)}{4\pi} L, \tag{S6}$$

which has dimension of length. From $\mathrm{Im}(\alpha)$ it is easy to obtain the absorbance coefficient $A(\omega)$ as [18,19]

$$A(\omega) = \frac{4\pi\omega}{c} \mathrm{Im}[\alpha^{2D}(\omega)], \tag{S7}$$

which is dimensionless.

From the polarizability it is possible to obtain the spontaneous emission rate γ^{sp} per unit energy, per unit surface. Following Ref. [20] and employing the Roosbroeck–Shockley relation [21], for a 2D material we define

$$\gamma^{sp} = \frac{(\hbar\omega)^3}{\pi^2 c^3 \hbar^4} \mathrm{Im}[\varepsilon(\omega)] L \, n_B(\omega) = \frac{4}{\pi} \frac{(\hbar\omega)^3}{c^3 \hbar^4} \mathrm{Im}[\alpha^{2D}(\omega)] \, n_B(\omega), \tag{S8}$$

where, given the exciton energy spectrum E_i^S, $n_B(\omega)$ is a Boltzmann weight that takes the nonzero value $n_B(\omega) = e^{-(\hbar\omega - E_0^S)/k_B T}$ only for $\hbar\omega = E_i^S$.

This emission rate is valid in this form when phonon-assisted transitions are neglected.

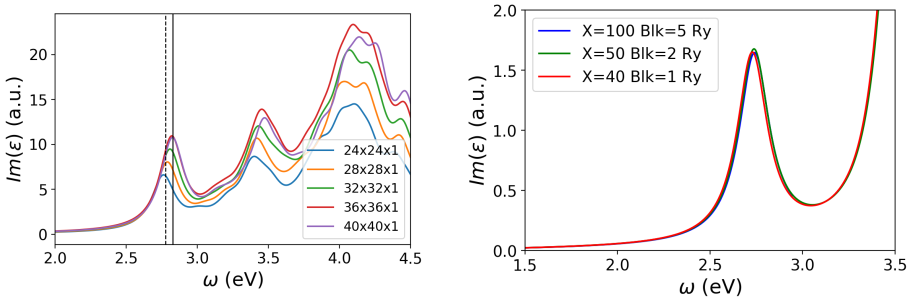

(a) (b)

Figure S5: Convergence tests on BSE calculations. (a) Variation of the absorption spectrum on different k-point grids. (b) Absorption spectra obtained on the coarse $24 \times 24 \times 1$ by varying the number of bands and the energy cutoff of the dielectric screening in the static-exchange approximation.

6 Average electron/hole densities

From the exciton state λ wavefunction $\Psi^{\lambda}(\mathbf{r}_e, \mathbf{r}_h)$, it is possible to obtain the average density of the electron (hole) by marginalizing the squared modulus $|\Psi^{\lambda}(\mathbf{r}_e, \mathbf{r}_h)|^2$ on the coordinates of the hole (electron)

$$\rho_e(\mathbf{r}_e) = \int d\mathbf{r}_h |\Psi^{\lambda}(\mathbf{r}_e, \mathbf{r}_h)|^2,$$
$$\rho_h(\mathbf{r}_h) = \int d\mathbf{r}_e |\Psi^{\lambda}(\mathbf{r}_e, \mathbf{r}_h)|^2,$$

where the integrals are extended to the whole space. These give the probabilities of finding the electron and hole of exciton λ at $\mathbf{r}_e$ and $\mathbf{r}_h$, respectively.

References

[1] P. Giannozzi, *et al.*, *Journal of Physics: Condensed Matter* **21**, 395502 (2009).

[2] P. Giannozzi, *et al.*, *Journal of Physics: Condensed Matter* **29**, 465901 (2017).

[3] J. P. Perdew, K. Burke, M. Ernzerhof, *Phys. Rev. Lett.* **77**, 3865 (1996).

[4] H. J. Monkhorst, J. D. Pack, *Phys. Rev. B* **13**, 5188 (1976).

[5] M. Wu, *et al.*, *Nanoscale* **10**, 11441 (2018).

[6] X. Chen, Z.-Z. Lin, M. Ju, *physica status solidi (RRL) – Rapid Research Letters* **12**, 1800102 (2018).

[7] M. R. Fiorentin, K. K. Kiprono, F. Risplendi, *Nanomaterials and Nanotechnology* **10**, 1847980420949349 (2020).

[8] A. Zobelli, C. P. Ewels, A. Gloter, G. Seifert, *Phys. Rev. B* **75**, 094104 (2007).

[9] H.-P. Komsa, T. T. Rantala, A. Pasquarello, *Phys. Rev. B* **86**, 045112 (2012).

[10] S. B. Zhang, J. E. Northrup, *Phys. Rev. Lett.* **67**, 2339 (1991).

[11] C. Freysoldt, *et al.*, *Rev. Mod. Phys.* **86**, 253 (2014).

[12] H.-P. Komsa, N. Berseneva, A. V. Krasheninnikov, R. M. Nieminen, *Phys. Rev. X* **4**, 031044 (2014).

[13] A. Marini, C. Hogan, M. Grüning, D. Varsano, *Computer Physics Communications* **180**, 1392 (2009).

[14] D. Sangalli, *et al.*, *Journal of Physics: Condensed Matter* **31**, 325902 (2019).

[15] F. Bruneval, X. Gonze, *Phys. Rev. B* **78**, 085125 (2008).

[16] L. Debbichi, O. Eriksson, S. Lebègue, *The Journal of Physical Chemistry Letters* **6**, 3098 (2015).

[17] X. Chen, Z.-Z. Lin, M. Ju, *physica status solidi (RRL) – Rapid Research Letters* **12**, 1800102 (2018).

[18] L. Yang, J. Deslippe, C.-H. Park, M. L. Cohen, S. G. Louie, *Phys. Rev. Lett.* **103**, 186802 (2009).

[19] M. Bernardi, M. Palummo, J. C. Grossman, *Nano Letters* **13**, 3664 (2013).

[20] F. Paleari, H. P. C. Miranda, A. Molina-Sánchez, L. Wirtz, *Phys. Rev. Lett.* **122**, 187401 (2019).

[21] W. van Roosbroeck, W. Shockley, *Phys. Rev.* **94**, 1558 (1954).
